# Visible Light Imaging: Clinical Aspects with an Emphasis on Medical Photography—a HIMSS-SIIM Enterprise Imaging Community Whitepaper

**DOI:** 10.1007/s10278-022-00584-0

**Published:** 2022-02-10

**Authors:** Cheryl A. Petersilge, Julie McDonald, Matthew Bishop, Laurence Yudkovitch, Caitlin Treuting, Alexander J. Towbin

**Affiliations:** 1 UPMC Department of Radiology, 200 Lothrop Street, UPMC Montefiore, Room NE 538, PA Pittsburgh, 15213 USA; 2 Hyland HealthCare, Westlake, OH 44145 USA; 3 Enterprise Solutions Architect - Imaging, UnityPoint Health, Bettendorf, IA USA; 4 iCAD, 98 Spit Brook Road, Suite 100, Nashua, NH 03062 USA; 5 Clinical Photography, Cincinnati Children’s Hospital, 3333 Burnet Avenue, MLC 5013, Cincinnati, OH 45229 USA; 6 Department of Radiology, Cincinnati Children’s Hospital, University of Cincinnati College of Medicine, 3333 Burnet Avenue, MLC 5013, OH 45229 Cincinnati, USA

**Keywords:** Enterprise imaging, Medical photography, Photodocumentation, Visible light imaging, Medical videography

## Abstract

Photodocumentation is a subset of visible light imaging and is an important growing segment of enterprise imaging. Medical videography is another subset of visible light imaging that shares many of the challenges of photodocumentation. Medical photographs are used to document clinical conditions, support diagnosis, guide, and document procedures and to enable collaboration among colleagues. They also play a significant role in patient engagement and are a mechanism for patients to share information with their provider without the need for a clinical office visit. The content of medical photographs raises issues for acquisition, management, storage, and access. Medical photographs may contain protected health information, and these images benefit from the standardized, secure processes inherent in any enterprise imaging program. The ability to securely acquire images on mobile, and sometimes personally owned devices, is a necessity. In addition to containing protected health information, photograph content can be sensitive or gruesome or the images may be used for forensic purposes. These types of images require additional protections. Access to these images should be role-based and auditable. To properly identify photographs and to convey information about their acquisition parameters new metadata requirements and mechanisms for its association with the imaging files are evolving. Institutional policies need to be developed to define the organization’s requirements for medical photography, including consent processes. Existing policies such as those defining the designated record set and legal health record should address the management of medical photography.

## Introduction

As enterprise imaging has advanced and embraced imaging modalities beyond the traditional Digital Imaging and Communications in Medicine (DICOM)-based imaging, recognition of the importance of medical photography and its supporting workflows has continued to grow. With the ubiquitous use of smart devices, medical photography has exploded. Any provider or patient can now generate a medical photograph at the point of care. The ability to acquire photographs enhances a provider’s documentation and improves communication along the entire care continuum. Patients have widely accepted the use of smart devices in the clinical setting, especially when hospital-owned devices are used [1–4]. Yet, most organizations are just beginning to address the challenges of properly labeling, securely managing, and providing appropriate access to the images.

This whitepaper is designed to provide a clinical perspective on medical photography within the framework of enterprise imaging. The enterprise imaging approach creates a safe and secure environment for all medical images ranging from DICOM-based studies to visible light images. It standardizes workflows, terminology, and image management; establishes appropriate access and viewing; and supports interoperability. In this whitepaper, we will define the terms related to visible light imaging and medical photography, identify key use cases for medical photography, and describe new challenges that must be solved for the successful incorporation of medical photography into an enterprise imaging ecosystem. A companion whitepaper will expand upon the technical elements of building a medical photography program. Many of the issues discussed in this whitepaper extended beyond medical photography and can be applied to medical videography.

The challenges of medical photography vary by country and within a country by local laws and regulations. Within the USA, there are variations among state law. The paper will focus on USA’s laws and regulations. International laws and regulations may be more restrictive than those in the USA.

## Terminology

To explore medical photography a series of shared definitions need to be established. The Healthcare Information and Management Systems Society (HIMSS) and the Society for Imaging Informatics in Medicine (SIIM) Enterprise Imaging Community Photodocumentation Workgroup have adopted the following definitions.

### Visible Light Imaging

According to the DICOM standard, visible light imaging refers to any image acquired by means of a camera or other sensors that are sensitive to visible or near visible light [5]. At the time of acquisition or post-acquisition, filters or other applications may be used to enhance these images and display information from nonvisible light energy [6]. As outlined in the DICOM standard visible light images are generated by multiple devices including:

- Endoscopy equipment
- Microscopes
- Ophthalmology equipment
- Digital or video cameras

### Photodocumentation

Photodocumentation is defined as the use of photography to record and document various aspects of patient management [7].

### Medical Photography

A broad category that includes clinical and non-clinical photography. The determination of whether a photodocument is clinical or non-clinical is based on the primary intent of the image.

#### Clinical Photography

A photograph obtained by, or shared with, a healthcare professional for the primary intent of supporting the care process. Clinical photography can be obtained for documentation of current state, to establish a diagnosis, or to guide and/or document a medical procedure. Within an enterprise imaging environment, clinical photography should be sent to the Enterprise Imaging archive and should be viewable using the enterprise viewer. This definition is independent of the person acquiring the image; thus, patient-generated images shared with a provider for the primary intent of diagnosis or care management are defined as clinical images [8]. According to The Joint Commission, clinical photographs do not require patient consent [9].

#### Non-clinical Photography

A photodocument obtained by, or shared with, a healthcare professional for a primary intent other than clinical care. Examples of non-clinical photography include marketing, patient identification, teaching, education, and research. In general, photographs that are never used for patient care should be segregated from clinical photographs and clinical viewing. Non-clinical photographs require patient consent for acquisition and use.

## Clinical Photography General Use Cases

In “A Foundation for Enterprise Imaging: HIMSS-SIIM Collaborative White Paper on Enterprise Imaging,” Roth et al. described the four major categories for enterprise imaging content: diagnostic imaging, procedural imaging, evidence imaging, and image-based clinical reports [10]. Clinical photography, while initially heavily focused on documentation of current state (evidence imaging), now spans all these content areas as reflected in the following use cases.

### Providers and Healthcare Organizations

Providers typically use clinical photography for one of four purposes: to document a diagnosis, to follow the progression of disease, to guide and/or document a procedure, or to consult colleagues. Clinical photography is desired in these scenarios as it is easy to obtain at the point of care, provides more objective evidence than a standalone written assessment, and can be rapidly transmitted to other providers [11]. Providers have found that a clear visual representation provides key supportive information when communicating with colleagues [12–14].

### Patients

#### Patient-Generated Photographs

Patient-generated photographs are gaining in importance as telehealth workflows expand. Photographs are a vehicle which allow a patient to share high-fidelity visual information with their provider, reducing the need for in-office visits between follow-up appointments. This sharing may occur during a live video visit when the provider desires to document a clinical finding, similar to the in-office process. Alternatively, in asynchronous, or store and forward, workflows the photograph may be the primary means of delivering information to the provider. A patient may send their provider a photograph of their condition with the intent of letting the provider evaluate the photograph and use the information to drive a care decision.

While patient-generated images can impact care delivery, in our experience, most patient-generated images are of lesser quality than provider-generated images. The decreased quality can be the result of lower quality cameras, non-standard lighting, non-standard color correction, and non-standard positioning. Because of these issues, we believe that these images should be labeled as patient-generated.

#### Patient Engagement

Clinical photographs are important tools for patient engagement. Patients report increased knowledge of their condition and greater involvement with their treatment plan when they are able to visualize their disease process through the use of photographs [4, 15, 16]. In one study, 88% of patients reported a belief that the quality of their care was improved through the use of photography [4]. In a study of patients with chronic wounds, more than 50% of patients indicated that photographs of their difficult-to-see wounds enhanced their involvement in the care process [15]. Images can be morphed over time to show healing in time-lapsed videos [17]. The technique of digital morphing has been useful in demonstrating healing in cases like frostbite, poisonous spider bites, and gunshot wounds.

## Clinical Photography’s New Challenges

Clinical photography presents new, unique challenges for Enterprise Imaging. Whether created by patients or providers, the photograph acquisition process is unstructured. Quality is inconsistent and difficult to monitor. Training is minimal, and acquisition devices are highly variable. Major concerns revolve around requirements for patient consent to acquire and use their images. Due to the rather casual nature of the image acquisition process, providers are often unaware of the security implications of clinical photography.

### Image Content

#### Protected Health Information

Photography and videography are imaging modalities that may create content that is currently designated as protected health information (PHI). In the initial proposed Health Insurance Portability and Accountability Act (HIPAA) Privacy Rule, all photographs were considered PHI [18]. However, in its final version, PHI only includes “individually identifiable health information,” when with this information, “there is a reasonable basis to believe the information can be used to identify the individual” [19]. Full-face photographs and any comparable images and biometric identifiers including finger and voice prints are included in the 18 types of patient identifiers [20]. As machine learning has progressed, many different facial features can be biometric. For example, the external ear, retina, and iris have all been used to identify an individual. Other skin-based features are also considered to be unique and have been used to help identify an individual. In this sense, tattoos, scars, and large or unique birthmarks should be considered identifying information. Some have proposed that the true test of “identifying information” is whether the patient can successfully recognize themselves in an image; if they can, the image is considered to contain PHI [21]. Many clinical photographs of the skin surface should be treated as if they contain PHI. Surface images that are highly magnified are less likely to contain PHI. Photographs of internal structures, such as those obtained during endoscopy, surgery, or pathologic assessment, are unlikely to contain PHI.

In the enterprise imaging ecosystem, maintaining the security of photographs is important, particularly when photographs are obtained using a provider’s personal device [22, 23]. Providers should only photograph patients using secure, institution-approved image capture devices and applications. Specifically, the photograph should not be stored in a photo library on the personal device. When stored on a personal device, the PHI risks are significant. The device may be lost or stolen leading to unauthorized access. The owner of the device risks inadvertent, unintended sharing of patient images. To further minimize risks, these images should be sent to an archive such as the enterprise imaging vendor neutral archive where they can be securely managed and accessed.

#### Sensitive Images

The definition of a sensitive image is subjective and ever-changing [3]. Many providers consider images of body parts that are typically covered by undergarments (such as the breasts, buttocks, and external genitalia) as sensitive. However, it should be noted that, while an organization may establish a definition for what constitutes a sensitive photograph, patients may have their own opinion regarding which of their photos should be considered sensitive. These differing opinions may relate to social differences including cultural, religious, sexual, or gender identity or mental health disorders such as body dysmorphic disorder.

As organizations wrestle with policies related to the acquisition, transmission, viewing, storage, and sharing of sensitive images [3, 24], some organizations may determine that acquisition of these images should not be permitted. However, we believe that a restrictive policy is inconsistent with the overall concept of photodocumentation. Instead of being restrictive, we advocate for policies and procedures that are protective, ensuring that appropriate consent is obtained, that strict permission-based controls are maintained, and that active auditing is performed to best protect patients.

#### Gruesome Images

Gruesome photographs can be described as those images which are bloody, gory, or violent [25]. Viewing these images may cause emotional distress and may elicit strong psychological reactions [26]. This emotional response has been studied in contexts such as the response of jurors and legal professionals who review gruesome photographs submitted as evidence and those images published by the media [26, 27]. Gruesome clinical photographs range from documentation of the injuries of a victim of a motor vehicle collision to intra-operative photographs. While most healthcare providers are trained to control their emotional reactions to such images, not all are desensitized. We advocate for gradated viewing options for providers set at the user level.

Now that patients are more engaged in the viewing of their medical information, access to gruesome images is a critical concern when building a medical photography program. We believe that patients should be warned before viewing a gruesome image. Ideally, gruesome images should be labeled with a text description of their contents. Patients will be better able to judge what they would like to see and to prepare themselves for viewing the image. For example, an intraoperative photograph of a tumor resection may be more tolerable to a patient than images obtained in the emergency room after an assault.

#### Medicolegal/Forensic Photography

Medical photographs play a critical role in the documentation of child abuse, elder abuse, and sexual assault [28–32]. One of the primary intents for acquiring these images is to provide forensic evidence in the prosecution of the offenders. Forensic images can provide documentation that impacts the course of the legal case. For example, the number of sexual assault charges filed, conviction rates, and average sentence length all increased when photographs are included in the evidence [33, 34]. Due to their sensitive nature, the way these photographs are handled requires special consideration. As potential evidence in legal proceedings, the chain of custody of these images must be impeccable [35, 36]. While these images may be considered part of the medical record, access must be strictly controlled with a clear record of the individuals who have viewed the images [35, 37–39]. Additionally, a duplicate copy is mandatory. In many institutions, forensic photographs are obtained and managed in systems distinct from the clinical systems.

Specific training is imperative to generating high-quality evidence. The quality of photographs impacts their evidentiary value [11, 40]. Additional concerns around these images include the format in which they are shared as evidence. The native format is preferred by courts [41, 42]. In at least one case, DICOM wrapping files precluded their use as evidence. A complete discussion of forensic photographs is beyond the scope of this manuscript.

### Consent

The respect for a patient’s privacy and psychological safety should be the primary concern for organizations as medical photography programs are implemented. Patient consent that addresses each potential use case for the medical photograph is the cornerstone of protecting the patient, the provider, and the organization. Unintended or misunderstood use of medical photographs can lead to patient’s anger, embarrassment, and mistrust for the patient/caregiver relationship and can have significant legal and financial ramifications [43, 44]. As stated by Kunde “of all forms of medical documentation, clinical photographs have the greatest potential to cause the patient distress” [45]. Risks increase if the use of a photograph is outside the scope of consent or patient’s understanding of the intended use.

The consent process must include a discussion about the intended use of the photograph, how the images will be stored, who will have access to view the images, and the associated security mechanisms that will be in place. The Joint Commission has an expectation of informed consent when photographing, creating video, digital, electronic, or audio of the patient and/or others who may be captured in that media when the images are “used for purposes other than the indemnification, diagnosis or treatment of the patient,” i.e., non-clinical photography [9]. As information blocking rules evolve, we anticipate that, by default, photographs may be shared unless the patient opts out. Each organization should address these situations in the appropriate policies. We also believe that vendors should be prepared to enact granular security measures based on the permission granted by the patient for a specific medical photography imaging study.

The Joint Commission recommends that organizations develop an informed consent policy or policies for medical photography. These policies should address circumstances under which images of patients may be obtained through recording devices (cameras, cell phones, etc.), how such images may be used, including clinical care, teaching, research, publication, and marketing, when informed consent is required and actions to prevent unauthorized access and use [9]. A “ladder of dissemination” was created by Devakumar to help guide the consent process [46]. The ladder describes different audiences that will view a photograph ranging from small audience teaching to having the photograph freely available on the internet.

Numerous factors including age, race, gender, and socioeconomic status influence patient’s willingness to provide consent for the variety of use cases [47]. There are also variations in comfort levels when the subject was deemed identifiable in the images [3]. Religion and personal dignity beliefs also play a role in determining a patient’s comfort level with the use of medical photographs [48]. Conferring with risk management and legal counsel to ensure compliance with state and federal laws and regulations is strongly recommended [9].

The major risk associated with all forms of medical photography—the inadvertent exposure of PHI—exists for the lifetime of the image. This risk is unlike many other healthcare risks which are event-related and time-constrained. For example, the risk of excessive bleeding related to a surgery is confined to the time during and immediately after the surgical procedure. The potential risk related to a medical photograph exists at all points in the image management process from acquisition through storage and viewing. These risks must be addressed and mitigated. A discussion of these risks is a key element of the consent process for photograph acquisition and use.

A critical element in the consent process is to have the patient understand that he or she has the right to decline to be photographed without any alteration in their care [13, 44]. However, if a physician believes that the care they deliver will be hampered when a patient declines to be photographed it is appropriate for that the physician to refer the patient to another provider [49]. It is also important for patients to know that they may withdraw consent at any point in time. The ability to withdraw consent is particularly important to acknowledge when photographing pediatric patients. The consent originally granted by the parents or caregivers may be withdrawn by the adult patient. Hospitals should include creating policies and procedures to detail what happens if a patient withdraws consent. Some organizations may determine that once an image is stored; it cannot be deleted. Thus, in this instance, withdrawal of consent may mean that the image remains archived but is not able to be viewed by providers. However, if the images have already been released to the public domain the process is irreversible.

As concerns about patients’ privacy intensify, the conditions for the publication of photographs in medical journals are becoming stricter. While written consent has been the default standard, now several journals require the patient to sign a journal-specific release [21]. This requirement may be difficult to meet for certain scenarios where the exact journal publishing a manuscript is not known at the time of photograph acquisition.

### Technique and Image Quality

In the medical setting, the acquisition of quality photographs is more complex than the simple act of taking photographs of friends and family to commemorate special occasions. In order to ensure high quality, consistent and accurate comparison between time points imaging parameters must be reproducible. Consideration must be given to numerous technical factors to allow the images to be acquired, captured, indexed, stored, managed, viewed, and shared through secure and efficient workflows.

In the years prior to the widespread use of portable smart devices, medical photography was highly controlled. Medical photography professionals generated most medical photographs. Dermatology and plastic surgery departments built photography studios. These studios provided a highly controlled environment resulting in reproducible high-quality images. Numerous reports on technique, patient positioning, and the number and types of views were published [50–55].

In today’s environment, the ubiquitous use of smart devices and digital cameras has led to an expansion of the number of medical photography use cases. The previously controlled studio environments are now replaced by photograph acquisition in any environment from the office to the emergency room. Many of the today’s providers are unaware of the technical requirements for high-quality photographs, established standard views, and positioning requirements. Numerous articles have been published in subspecialty journals to help communicate the need for optimal conditions for photodocumentation outside of a studio [13, 56–62].

Patient expectations will increase as medical photography becomes part of routine care. As stated by Lakdawala “as digital imaging becomes more accepted as a diagnostic tool, much like routine labs or radiological studies, patients will come to expect a greater level of accuracy and reliability in these images” [44].

### Image Labeling

Metadata requirements for clinical photographs have yet to be defined. Appropriate metadata serves a multitude of purposes, and image value is increased when associated with rich metadata. Metadata supports efficient technical and clinical workflows. Items such as “body part imaged” will serve to identify all relevant images regardless of image format [63]. This correlation across image formats enables the recognition of relevant comparison studies. Technical standardization will be supported by meshing metadata elements for photographs with existing enterprise imaging standards. This technical standardization supports consistent content and coding systems. Knowledge about device type and acquisition parameters is necessary to achieve consistent and reproducible image quality. The ability to reproduce an image’s color profile is essential when comparing photographs. Ideally, this information should be stored in the metadata. In addition to supporting efficient clinical and technical workflows and standardization, we believe that many of these elements will impact the future value of the images as enterprise viewers become more efficient at displaying images, and their relevant comparative studies and as artificial intelligence applications become more robust.

The HIMSS-SIIM Enterprise Imaging Community Photodocumentation Workgroup has identified the types of information which may be important to store in the metadata. Currently, this information may not be available from all devices and applications. The purpose of this list is to be provocative and aspirational. It should spur discussion within each organization as they select technology and establish workflows and policies for their medical photography programs. The technical aspects of capturing and storing this metadata will be included in the companion technical whitepaper and are beyond the scope of this clinical whitepaper. The list of proposed metadata elements is summarized in Table 1. The list is organized into four levels of metadata which follow the four levels of the Encounters Based Workflow [64].

**Table 1:** Clinical photography metadata

Category	Elements
Patient level	Name Medical record number Date of birth Sex
Encounter level	(Aligns with study in DICOM) Clinical indication Date of event Event identifier Event description Geographic location Ordering provider
Procedure level	(Aligns with series in DICOM) Operator Acquisition device (manufacturer, model) Acquisition device type Device ID Body part(s) Laterality
Object level	Date/time of object creation Gruesome/sensitive Consent to share (yes/no) Unique object-level identifier Camera viewpoint Filters Flash (yes/no) Color profile Color depth Pixel depth Resolution Camera exposure Single object/series Video or still Distance Focal plane File format Standard view (yes/no) Body part Laterality

### Image Access and Viewing

Each institution must establish its own parameters for image access. To facilitate those discussions, the HIMSS-SIIM Enterprise Imaging Community Photodocumentation Workgroup has developed a series of role-based guidelines for image access. These guidelines can be used to shape an enterprise imaging program as it seeks to ensure access to all imaging information across the continuum of care. This access must be balanced by concerns for patient privacy. Additionally, as healthcare practices evolve, providers are no longer the sole consumers of images. Patients are becoming well-established as consumers of their own healthcare information [65]. The increase in patient engagement will be heightened with the United States 21st Century Cures Act [66]. Thus, patient viewing of images must be accounted for as enterprise imaging programs develop.

Example uses cases and their guidelines for access are outlined in Table 2. These guidelines are designed to inform each institution’s discussion and to convey to vendors the desired features. The solutions to achieve these types of image access are still in their infancy. This Table is intended to prompt discussion at the institution level and innovation amongst the vendors.

**Table 2:** Guidelines for role-based access to clinical photographs. The access guidelines are organized by the various image contents that have been discussed: sensitive, gruesome, and medicolegal. The guidelines apply to image access via the enterprise viewer based on the assumption that this viewer will be used by an organization to share these photographs with providers and patients. If other mechanisms are used to provide access to these images, the same considerations should apply. Access proxy refers to parents, guardians, durable medical power of attorney, or any individual with legal power to make decisions for a patient. Image sharing includes all mechanisms for distributing images to external providers or healthcare organizations. Access type defines the type of warning or restrictions placed on image access via the enterprise image viewer. These restrictions should not violate state or federal laws and should not impede care. The default setting for access type is unrestricted access. As the individual interacting with the system providers are responsible for setting access type. The guidelines recognize that patients will interact with their images and should have a voice in identifying image content as sensitive or gruesome

**Image content**	Who sets access type	Access—provider	Access—patient	Access—proxy	Sharing	Access type
General use	Default	All	All	All	All	Open
Forensic and evidentiary photos; research	Default	None or break-the-glass or request permission functionality	None	None	None	Sequestered
Sensitive images	Provider	Role-based	All	Per patient	Per clinical needs, patient preference, release of information policies	Viewer warning or role-based access with break-the-glass or request permission functionality
Gruesome photographs	Provider	All	All	Per patient	Per clinical needs	Viewer warning to patient, provider or both

### Health Information Management

Any information generated about a patient during a clinical encounter is considered part of the patient’s medical record, and we believe that clinical photographs should be included as part of the medical record. Medical records must be managed in accordance with institutional policy as well as by state and federal regulations. Each healthcare organization will need to have its own policies to meet these federal and state requirements.

#### Legal Health Record and Designated Record Set

The legal health record (LHR) and the designated record set (DRS) are defined subsets of a patient’s medical record. The contents of the LHR and DRS are not defined at the federal or state level [67]. Each organization is responsible for determining what information is included in each of these records. The legal health record is the official business record of an organization and includes information about the clinical care of a patient and defines the information that is released when a patient’s medical records are requested [67]. The DRS was introduced in the HIPAA Privacy Rule [19]. It is more expansive than the LHR, and it contains both clinical information and business information such as billing records. Patients have the rights to access, request amendments, and submit request to limit disclosure of the DSR, and they have a right to know who has accessed their information.

Clinical photograph acquisition is an integral part of the care process and is used to make decisions about an individual. It thus meets the criteria outlined in the Privacy Rule for inclusion in the DRS [19], and organizations such as the American Health Information Management Associate (AHIMA) recommend that clinical photographs be considered part of the DSR [67]. Inclusion of photographs in the LHR is more variable. These requirements may differ from other types of imaging which are considered source clinical data and not traditionally part of the LHR or DRS [67]. Organizations should ensure that all their policies are internally consistent.

#### Release of Information

Photographs containing identifying information will be subject to the same regulations as any other PHI. PHI may be released without authorization when needed for treatment, payment, and healthcare operations including release to any provider involved in the care of the patient regardless of whether they are in the same healthcare organization [19]. While the Final Rule states that consent is not required when information, including images, is released for clinical purposes, some states have laws requiring authorization [19]. PHI may be released to the individual without authorization. Patients may request restricted release of portions of their DSR. Institutions, according to their policies, must evaluate these requests [68]. Due to the sensitive nature of many photographs, consideration may be given to not including photographs in the initial release of information.

### Lifecycle Management

According to the Privacy Rule, information may be defined as part of the DSR regardless of how that information is retrieved [19]. The HIMSS-SIIM Enterprise Imaging Community Photodocumentation workgroup recommends that all images be stored using good information technology practices with redundant storage, high availability, and HL7 interfaces to allow for metadata updates. Images should be stored whenever they are used for medical decision-making. As part of the medical record, life cycle management policies should be developed for clinical photographs.

### Ownership and Copyright

The rights of ownership and copyright for medical photographs are complicated and reflect the complexity associated with the EMR. The following information is available in the literature. This information should not be considered legal advice. Whenever a concern is raised appropriate legal counsel should be obtained. There are 21 states that have determined that the physical medical records are the property of the hospital or physician. New Hampshire states that the patient owns the information in his or her medical record [69]. Ownership is not clearly defined for the other states.

Ownership of the medical record is intermingled with copyright. Copyright “is defined as the exclusive legal right to reproduce, publish, sell, or distribute the matter and form of something (such as a literary, musical, or artistic work)” [70]. “Copyright is an exclusive right that is automatically assigned to the creator at the moment work is created” [71]. Copyright belongs to the individual who captures the image [72]. However, according to the US Copyright Office, medical imaging is not copyrightable. This judgment was based on medical imaging and works produced by a machine or mere mechanical process without creative input [73]. This judgment certainly applies to DICOM-based imaging such as radiology and cardiology. While the document does not specifically address medical photography, since medical photographs are not generated for creative purposes and are acquired via a standard process, we believe that clinical photographs are likely to be included in the copyright exclusion for medical imaging.

When an individual is photographed, they control the rights to those images [74]. With medical photographs, a physician cannot capitalize on use of the photos without the patient’s explicit permission” [75]. There are many case examples of litigation surrounding unauthorized use of a patient’s photographs [76]. This concept highlights the need for appropriate consent, based on the intended use of the photograph, as described earlier.

### Security

The HIPAA Security Rule demands that any PHI, including images, must be securely transmitted from the acquiring device to the archive. Additionally, storage locations must be secure and supported by necessary disaster recovery policies. The HIPAA Security Rule specifically addresses Individually Identifiable Health Information that is an electronic format. This rule “specifies a series of administrative, technical, and physical security procedures for covered entities to use to assure the confidentiality, integrity and availability of ePHI” [77]. The HIPAA Security Rule requires an organization to have the proper policies and procedures to ensure that access to PHI is only granted to the appropriate individuals and to ensure the security of that information. In the technical safeguards included in the rule, the covered entity is required to ensure access is only granted to authorized persons, that an audit trail exists to identify those persons who have accessed PHI. Additionally, it outlines the requirements for the means by which photographs are transmitted and stored; the data must be maintained in a way that it is not altered or destroyed. Inappropriate treatment of certain images can compromise the value of photographs as legal evidence. An enterprise imaging program provides the infrastructure and workflows needed to meet these requirements. The technical solutions to fulfill these requirements are described in part II of this whitepaper.

#### Patient-Generated Photographs

Concerns about the transmission of photographs from patient to provider have been raised. According to HIPAA, when information is generated by an unregulated actor (not a covered entity) the information is unregulated [68]. When that information is obtained by a regulated entity (covered entity) and it meets the criteria, that information becomes PHI. This process would imply that patient-generated photographs containing identifying information become classified as PHI when received by the organization, not before receipt.

## Conclusions

The incorporation of clinical photographs into the comprehensive longitudinal medical record via enterprise imaging is an evolving pathway. This clinical whitepaper has explored many of the issues an organization must address as they begin this journey. These issues include issues of patient consent, management of sensitive and gruesome images, and proper metadata among several others. Many of the recognized challenges do not yet have technical solutions. Continued discussion and awareness among providers, imaging informaticists, and vendor colleagues will enhance the available solutions for incorporation of clinical photographs into an enterprise imaging program.
